# Hybrid grading systems improve progression risk stratification in non‐invasive papillary urothelial carcinoma and support a histomorphological continuum of disease progression

**DOI:** 10.1111/his.70260

**Published:** 2026-08-10

**Authors:** Jane K Nguyen, Marcello P Toscano, Jianbo Li, Sean R Williamson, Christopher G Przybycin, Chia‐Sui Kao, Jonathan L Myles, Kristýna Pivovarčíková, Marián Švajdler, Marek Brousil, Mohamed Eltemamy, Laura Bukavina, Venkatesh Krishnamurthi, Christopher Weight, Rebecca A Campbell, Nima Almassi, Reza Alaghehbandan

**Affiliations:** ^1^ Robert J. Tomsich Pathology and Laboratory Medicine Institute Cleveland Clinic Cleveland Ohio USA; ^2^ Department of Quantitative Health Sciences Cleveland Clinic Cleveland Ohio USA; ^3^ Sikl's Department of Pathology, Medical Faculty in Pilsen Charles University Pilsen Czech Republic; ^4^ Bioptical Laboratory Pilsen Czech Republic; ^5^ Glickman Urological and Kidney Institute Cleveland Clinic Cleveland Ohio USA

**Keywords:** grading, histomorphological continuum, hybrid grading, non‐muscle‐invasive bladder cancer, papillary urothelial carcinoma, prognostic stratification

## Abstract

**Objective:**

Histological grading is a key predictor of progression in non‐muscle‐invasive bladder cancer (NMIBC). The WHO 1973 three‐tier system is limited by heterogeneity within the G2 category, whereas the WHO 2004/2016 binary classification reduces prognostic resolution by grouping biologically diverse tumours into broad low‐grade (LG) and high‐grade (HG) categories. We aimed to evaluate the prognostic performance of hybrid grading systems compared with WHO 2004/2016, while further characterizing the histomorphological continuum of non‐invasive papillary urothelial carcinoma.

**Methods:**

Non‐invasive papillary urothelial carcinomas (2012–2023; *n* = 472) were reviewed and reclassified by two urological pathologists according to WHO 2004/2016, hybrid three‐tier and four‐tier systems, and an investigational five‐tier framework (Grades I, IIA, IIB, IIIA, IIIB) integrating architectural, cytological and proliferative features. Primary endpoints were recurrence, grade progression and T1+ stage progression, assessed using ROC analysis, Cox proportional hazards regression and model‐performance metrics (AIC, BIC, C‐index).

**Results:**

Increasing morphological atypia was associated with larger tumour size, greater multifocality and progressively higher progression risk, with events concentrated in Grades IIIA and IIIB. All systems showed limited recurrence discrimination (C‐index 0.54–0.56). For T1+ progression, discrimination improved with increasing stratification (AUC 0.754 for WHO 2004/2016 vs. 0.815, 0.828 and 0.827 for the hybrid three‐tier, four‐tier and five‐tier systems, respectively; all *P* < 0.001). The Hybrid 3‐Tier model achieved the most favourable AIC and BIC, while the five‐tier model yielded the highest C‐index (0.818).

**Conclusions:**

Hybrid grading systems improve progression‐risk stratification in non‐invasive papillary urothelial carcinoma compared with WHO 2004/2016, with most benefit achieved by the Hybrid 3‐Tier framework. These findings support the concept of a histomorphological continuum of progression risk and favour continued refinement of biologically informed hybrid grading for NMIBC risk stratification.

## Graphical abstract

Hybrid grading systems significantly improve progression‐risk stratification in non‐invasive papillary urothelial carcinoma compared with the WHO 2004/2016 binary classification, with most benefit achieved by a Hybrid 3‐Tier framework. These findings support the existence of a histomorphological continuum of progression risk within the conventional high‐grade spectrum.

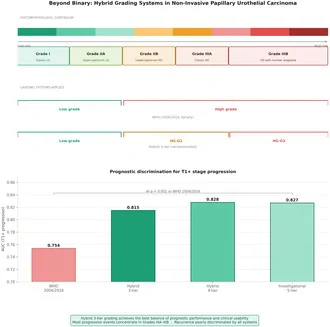

**Abbreviations**
AIC: Akaike information criterion
AUA: American Urological Association
BIC: Bayesian information criterion
CI: confidence interval
CIS: carcinoma in situ
EAU: European Association of Urology
HG: high‐grade
HR: Hazard ratio
LG: low‐grade
NMIBC: non‐muscle‐invasive bladder cancer
ROC: receiver operating characteristic
SD: standard deviation
WHO: World Health Organization

## Introduction

Histological grade is an important predictor of progression in non‐muscle‐invasive bladder cancer (NMIBC), although it is less relevant for predicting tumour recurrence.^1^, ^2^, ^3^, ^4^, ^5^ Over the last century, multiple grading systems have been proposed to improve risk stratification and guide clinical management of NMIBC.^4^, ^6^, ^7^, ^8^, ^9^, ^10^, ^11^, ^12^, ^13^ Among these, the two most widely adopted systems worldwide are the World Health Organization (WHO) 1973 three‐tier classification (grade [G]1, G2 and G3) and the WHO 2004/2016 classification (papilloma, papillary urothelial neoplasm of low malignant potential [PUNLMP], low grade [LG] and high grade [HG]).^14^, ^15^, ^16^ The American Urological Association (AUA) endorses the WHO 2004/2016 system, whereas the European Association of Urology (EAU) recommends dual grading using both the WHO 1973 and WHO 2004/2016 schema.^17^, ^18^, ^19^

The WHO 1973 grading system was limited by poorly defined histological criteria, resulting in substantial interobserver variability and accumulation of most tumours within the heterogeneous intermediate G2 category. This limitation prompted development of the revised WHO/ISUP classification in 1998, which introduced the categories of PUNLMP, LG and HG urothelial carcinoma.^8^ The system was subsequently adopted by the WHO in 2004 and retained in later updates.^15^, ^16^, ^20^ Compared with WHO 1973, the WHO 2004/2016 system provides clearer morphological definitions and improved reproducibility; however, it was introduced without robust evidence demonstrating superior prognostic performance.^21^

The WHO 1973 and WHO 2004/2016 systems are not directly comparable because they rely on fundamentally different conceptual frameworks and partially overlapping grade categories. Evidence evaluating these systems demonstrates that neither provides optimal prognostic discrimination for recurrence or progression.^1^, ^2^, ^22^

While WHO 1973 generally demonstrates slightly improved stratification of progression risk, the WHO 2004/2016 model compresses biologically heterogeneous tumours into overly broad categories, particularly within the HG group, thereby limiting prognostic resolution and obscuring clinically meaningful biological differences among tumours with divergent behaviour and variable response to intravesical therapy such as BCG.^22^ This limitation has become increasingly relevant as accumulating molecular, proliferative and clinicopathological data demonstrate substantial biological diversity within conventionally defined HG tumours, including differences in progression risk, genomic instability, molecular subtype and treatment responsiveness.^23^, ^24^

Increasing evidence suggests that hybrid grading systems may better capture this histomorphological continuum by integrating strengths of both WHO classifications.^1^, ^25^ Over the last decade, a Hybrid 3‐Tier grading system has been proposed that retains the WHO 2004/2016 LG category while subdividing the clinically heterogeneous HG group into HG/G2 and HG/G3 according to WHO 1973 principles.^1^, ^4^, ^25^, ^26^, ^27^, ^28^ Importantly, these hybrid systems should not be viewed merely as alternative classification schemes, but rather as biologically informed refinements designed to better reflect the underlying morphological continuum of papillary urothelial carcinoma. By preserving the reproducibility advantages of binary grading (LG vs. HG) while restoring clinically meaningful stratification within the HG category, hybrid approaches may improve identification of patients at increased risk for progression and reduce the potential for under‐ or overtreatment associated with uniform management of all HG lesions.

However, direct comparative validation between conventional and hybrid grading systems remains limited, and the full histomorphological continuum of non‐invasive papillary urothelial carcinoma has not been comprehensively characterized. Existing grading systems also rely predominantly on qualitative morphological descriptors with limited integration of more objective or semi‐quantifiable histological parameters, resulting in persistent ambiguity in defining biologically meaningful transitions between grade categories. Therefore, the aim of this study was to evaluate the prognostic performance of hybrid grading systems in comparison with WHO 2004/2016 classification, while further investigating the histomorphological continuum of non‐invasive papillary urothelial carcinoma using a structured five‐tier investigational framework incorporating more refined architectural, cytological and proliferative features, including mitotic activity, apoptotic activity, nuclear size, pleomorphism, chromatin characteristics and degree of architectural disorganization. This framework was not intended as a practical grading system for routine clinical use, but rather as a research tool to better characterize tumour heterogeneity, refine existing histological grading criteria, and identify biologically and clinically meaningful stratification thresholds that may support refinement towards a more biologically coherent and clinically practical hybrid grading model.

## Materials and methods

This study was approved by the Institutional Review Board of the Cleveland Clinic Foundation. We performed a retrospective review of all institutional non‐invasive papillary urothelial carcinomas at the Cleveland Clinic between 2012 and 2023.

### Cohort Selection

The Cleveland Clinic PIRO (Pathology Information Retrieval Optimizer),^29^ an institutional web‐based platform enabling efficient text searches of diagnostic pathology archives, was searched over a 10‐year period (2012–2023) using the following keywords: “bladder”, “non‐invasive”, “urothelial” and “carcinoma”. All transurethral resections (TURBT)/biopsies of bladder diagnosed as non‐invasive papillary urothelial carcinoma were retrieved, and all available histological slides were reviewed by two urological pathologists (J.K.N. and R.A.), with the original diagnoses and clinical outcomes masked, to confirm the diagnosis and grade the tumours.

Clinicopathological inclusion criteria were aligned with AUA NMIBC risk categories, such that LG tumours were limited to AUA low‐risk disease, while HG tumours comprised AUA intermediate‐ and high‐risk disease.^18^ Patients with very‐high risk features including BCG unresponsiveness, lymphovascular invasion, carcinoma in situ (CIS) and prostatic urethral invasion were excluded from the study. Further, cases containing recognized WHO histological subtypes (including micropapillary, plasmacytoid, nested, sarcomatoid and other subtypes) were excluded to permit evaluation of grading within conventional non‐invasive papillary urothelial carcinoma without the confounding prognostic effects of subtype histology. The resulting cohort represented a clinically curated spectrum of conventional non‐invasive papillary urothelial carcinoma intended to facilitate evaluation of histomorphological and biological heterogeneity across the LG to HG continuum, independent of established very‐high‐risk features. We also excluded re‐staging specimens, defined as any follow‐up TURBT performed within the first 90 days post the initial diagnosis.

Electronic medical records were reviewed to obtain the following demographic, clinical and pathological information: patient age at initial resection, sex, date of resection, tumour size (based on cystoscopy report), tumour focality, details of follow‐up resections, date of recurrence, date and type of progression (grade vs. stage) and occurrence of metastases.

### Pathology Re‐Review: Diagnostic Criteria for Tumour Grading

Tumours were graded using a structured, five‐tier system (I, IIA, IIB, IIIA and IIIB) (Table 1) designed to integrate and extend established criteria from the WHO 1973 and 2004/2016 classifications, as well as previously described hybrid grading schemes.^4^, ^7^, ^14^, ^15^, ^16^, ^30^ Grading criteria for each tier were established based on integration of WHO and previously published hybrid grading parameters before slide review, with additional morphological features incorporated during the study when consistently identified as reproducible histological patterns. Tumour grading was conceptualized as a biological continuum and assessed using a composite evaluation of architectural organization, cytological atypia and proliferative activity, with the aim of capturing the full morphological spectrum between conventional low‐ and high‐grade categories.

**Table 1:** Histomorphological features of the investigational five‐tier grading framework and corresponding who/hybrid system equivalents

Grade	Architecture	Cytology	Proliferation	Additional features	Approximate correspondence^a^
Grade I *Classic LG*	Preserved polarity and cellular cohesion; possible retained umbrella cells; basal palisading, focal epithelial streaming	Small uniform nuclei (~2× lymphocyte size); fine chromatin; smooth nuclear membranes; inconspicuous nucleoli; low N:C ratio	Rare mitotic figures (<2/10 HPF), mainly confined to basal layer; rare apoptosis	Basal palisading, nuclear grooves and focal clear cytoplasm may be present	WHO 2004/2016 LG; WHO 1973 Grade 1
Grade IIA *Upper‐spectrum LG*	Predominantly LG architecture with focal (<5% of tumour volume) architectural disarray, mild loss of polarity and focal discohesion; otherwise orderly background	Focal nuclear enlargement (~3–4× lymphocyte size); hyperchromasia; no pleomorphism	Focally increased mitotic activity (2–4/10 HPF), including occasional suprabasal mitoses; associated apoptotic clusters	Umbrella cells focally reduced; lacks diffuse atypia and proliferative activity of HG lesions; excludes tumours with any bona fide HG morphology^b^	Transitional upper‐spectrum LG; no direct WHO equivalent
Grade IIB *Lower‐spectrum HG*	Predominantly disordered architecture with papillary fusion and branching; reduced cellular cohesion; marked reduction of umbrella cells	Enlarged hyperchromatic nuclei (~3–4× lymphocyte size); mild‐to‐moderate pleomorphism; emerging nucleoli	Frequent mitotic figures (~5–7/10 HPF), extending above the basal layer; apoptotic aggregates; occasional focal necrosis	Represents the lower end of the HG morphological spectrum	Hybrid HG‐G2; lower‐spectrum WHO 2004/2016 HG
Grade IIIA *Classic HG*	Marked architectural disorganization; loss of polarity; fused papillae and/or focal solid growth; umbrella cells absent	Large pleomorphic nuclei (>5× lymphocyte size); diffuse hyperchromasia; prominent nucleoli; loss of nuclear grooves	Frequent mitotic figures, including atypical forms; frequent apoptosis	Fully developed conventional HG urothelial carcinoma morphology	WHO 2004/2016 HG; WHO 1973 Grade 3; Hybrid HG‐G3
Grade IIIB *HG with Nuclear Anaplasia*	Papillary/solid growth pattern; complete loss of polarity and cellular cohesion	Extreme cytological atypia; marked nuclear anaplasia (nuclei >4× adjacent tumour nuclei); profound pleomorphism	Numerous atypical mitotic figures; extensive apoptosis; necrosis common	Represents the extreme end of HG cytological atypia	Anaplastic end of HG spectrum; no direct WHO equivalent

HG, high grade; HPF, high‐power field; LG, low grade; N:C, nuclear‐to‐cytoplasmic; WHO, World Health Organization.

^a^ Approximate correspondence is provided for conceptual comparison only and does not imply direct equivalence between grading systems.

^b^ Grade IIA does not correspond to the WHO 2022 category of predominantly low‐grade papillary urothelial carcinoma with a focal (<5%) high‐grade component. Any tumour containing unequivocal conventional high‐grade morphology, regardless of extent, was assigned to the high‐grade spectrum (Grade IIB or IIIA).

Architectural assessment included papillary configuration (delicate vs. fused/branching, either exophytic or endophytic), degree of cellular cohesion and preservation or loss of polarity, as well as the presence or absence of umbrella cells. Cytological features evaluated included nuclear size (relative to lymphocytes), chromatin pattern, nuclear membrane contour, nucleolar prominence and degree of pleomorphism. Additional cytological features incorporated from hybrid systems included the presence of nuclear grooves and cytoplasmic characteristics (e.g. clear cytoplasm in LG lesions). Proliferative activity was assessed semi‐quantitatively in representative hotspot regions, including mitotic frequency, distribution of mitotic figures (basal vs. full‐thickness), presence of atypical mitotic figures, and the presence and extent of apoptotic aggregates and tumour necrosis. Using this structured, schematic approach, tumour grading was conceptualized as a biological continuum, allowing the full spectrum of morphological variation to be captured and compared as follows:

Grade I (classic LG) (Figure 1A–D) was defined by delicate papillae with preserved polarity, cohesive urothelial cells and retained umbrella cells, often with epithelial streaming. Cytological atypia was minimal, characterized by small uniform nuclei approximately twice the size of lymphocytes, fine chromatin, smooth nuclear membranes, inconspicuous nucleoli and a low nuclear‐to‐cytoplasmic ratio. Mitotic activity was rare (<2 mitotic figures/10 HPF) and mainly confined to the basal layer, with only occasional apoptosis. Additional supportive features included basal palisading, nuclear grooves and focal clear cytoplasm.

**Figure 1:**
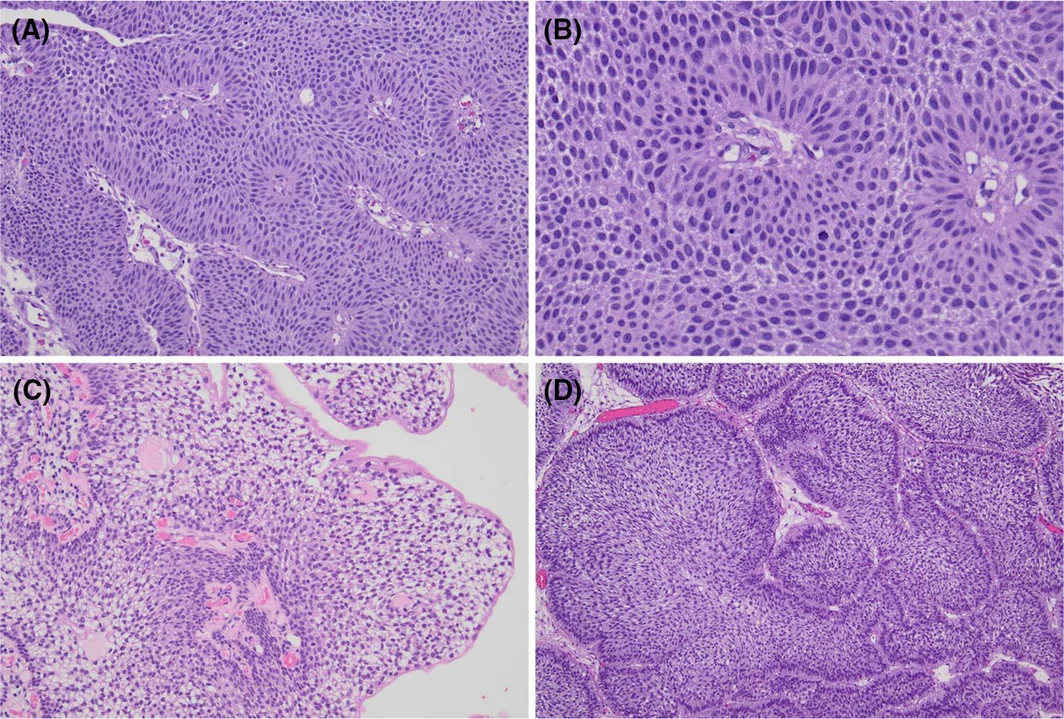
Grade I (Classic Low‐Grade) papillary urothelial carcinoma. **(A)** Low‐power view demonstrating delicate papillae with preserved cellular polarity and cohesion. **(B)** High‐power view showing small uniform nuclei with fine chromatin, smooth nuclear membranes and rare mitotic activity. **(C)** Clear cytoplasmic features. **(D)** Basal palisading and focal epithelial streaming.

Grade IIA (upper‐spectrum LG) (Figure 2A,B) consisted of predominantly LG tumours containing focal areas involving <5% of the tumour volume with early architectural and cytological deviation from classic LG morphology. These foci demonstrated focal architectural disarray, mild loss of polarity and focal discohesion within an otherwise orderly background. Cytological atypia included nuclear enlargement (approximately 3–4× lymphocyte size), hyperchromasia, with no pleomorphism. Proliferative activity was focally increased (2–4 mitotic figures/10 HPF), with mitotic figures extending above the basal layer and associated apoptotic clusters. Umbrella cells were focally reduced. Importantly, these limited foci lacked the diffuse proliferative activity and widespread cytological atypia characteristic of Grades IIB and IIIA (Figure 3A,B). It should be noted that Grade IIA does not correspond to the WHO 2022 category of predominantly LG papillary urothelial carcinoma with a focal (<5%) HG component (Figure 3C,D).^20^ In our framework, the presence of any unequivocal conventional HG morphology resulted in assignment to the HG spectrum (Grade IIB or IIIA), irrespective of extent, based on evidence that grade‐heterogeneous tumours represent biologically progressive lesions.^31^, ^32^, ^33^ Accordingly, Grade IIA was restricted to tumours lacking bona fide HG differentiation and exhibiting only limited transitional features between conventional LG and HG morphology.

**Figure 2:**
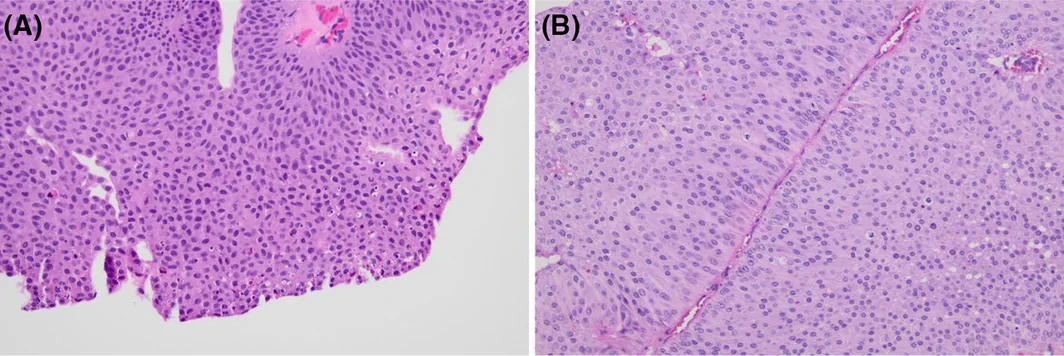
Grade IIA (Upper‐Spectrum Low‐Grade) papillary urothelial carcinoma. **(A)** Predominantly low‐grade architecture with focal areas of architectural disarray, mild loss of polarity and a focal area with a collection of apoptotic bodies. **(B)** Higher power view demonstrating mild nuclear enlargement with occasional mitotic figures.

**Figure 3:**
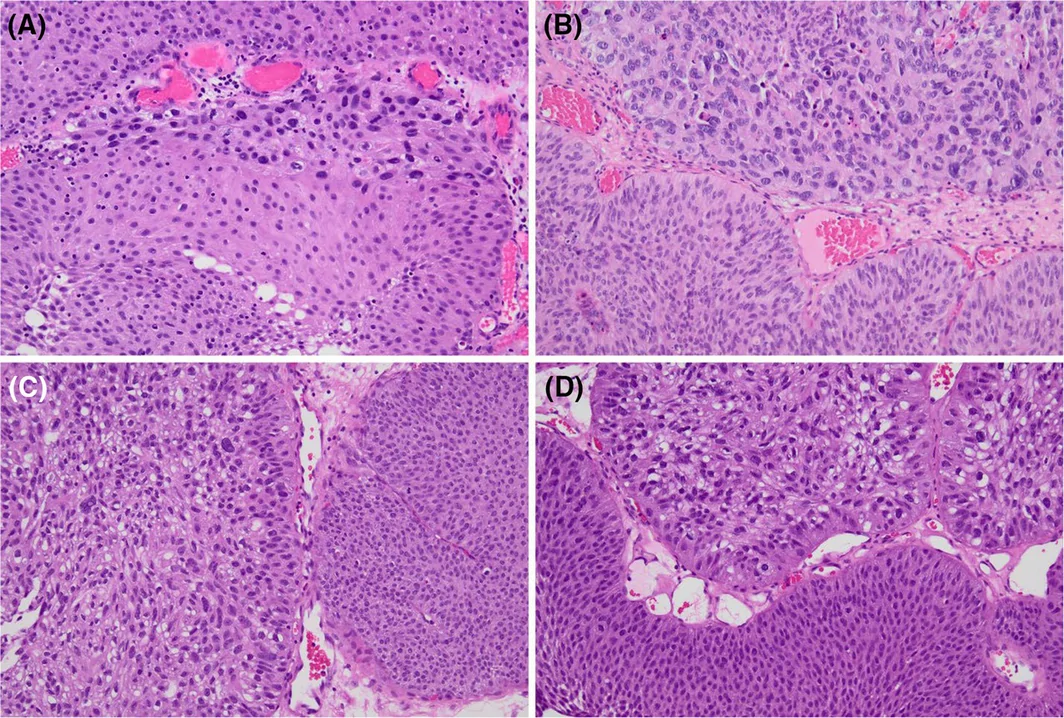
Distinction between Grade IIA and the WHO 2022 category of predominantly low‐grade papillary urothelial carcinoma with focal high‐grade component. **(A–D)** Examples illustrating the focal high‐grade features with proliferative activity and widespread cytological atypia characteristic of Grades IIB and IIIA, contrasting with adjacent/background low‐grade areas. In the present framework, any tumour containing bona fide high‐grade morphology, irrespective of extent, was assigned to the high‐grade spectrum (Grade IIB or IIIA) rather than Grade IIA. [Colour figure can be viewed at wileyonlinelibrary.com]

Grade IIB (lower‐spectrum HG) (Figure 4A–D) demonstrated predominantly disordered architecture with papillary fusion and branching, reduced cellular cohesion and marked reduction of umbrella cells. Cytological atypia was more pronounced, with enlarged hyperchromatic nuclei (approximately 3–4× lymphocyte size), mild–moderate pleomorphism and emerging nucleoli. Mitotic activity was frequent (≥3/HPF or approximately 5–7 mitotic figures/10 HPF), often accompanied by apoptotic aggregates and occasional focal necrosis. These tumours represented the lower spectrum of HG morphology.

**Figure 4:**
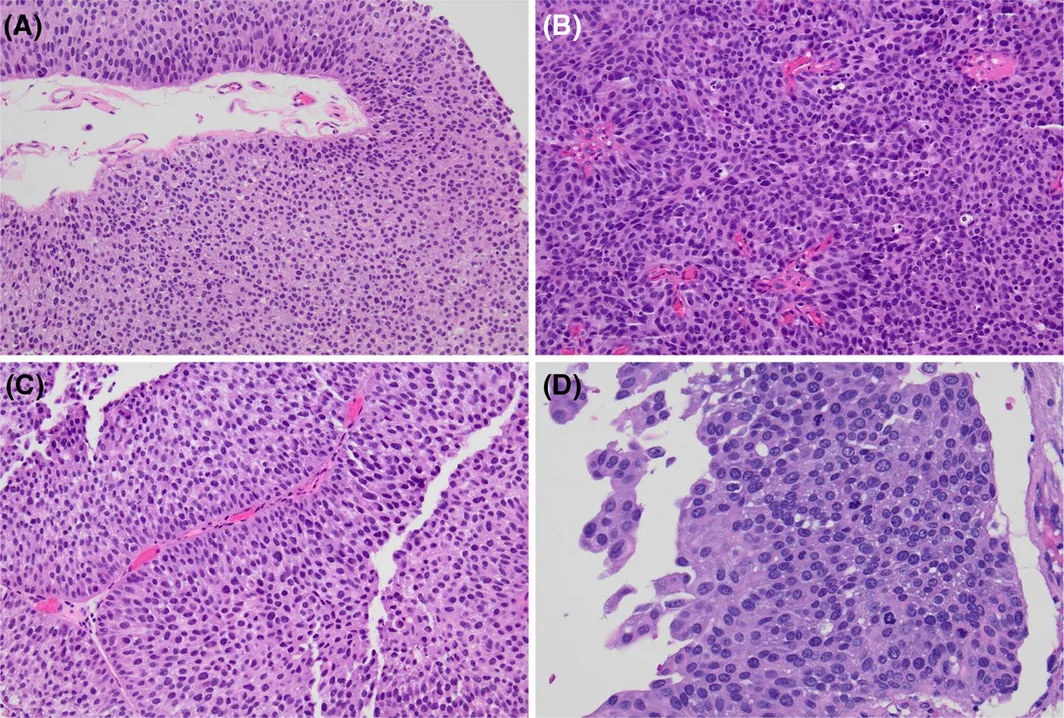
Grade IIB (Lower‐Spectrum High‐Grade) papillary urothelial carcinoma. **(A)** Disordered architecture with loss of polarity and frequent mitosis. **(B)** Diffuse nuclear enlargement and hyperchromasia with mild‐to‐moderate pleomorphism and occasional mitosis. **(C)** Papillary fusion and branching with disorganized urothelial proliferation and increased mitotic activity. **(D)** High‐power view demonstrating enlarged hyperchromatic nuclei, emerging prominent nucleoli and frequent mitotic figures, representing the lower end of the high‐grade morphological spectrum. [Colour figure can be viewed at wileyonlinelibrary.com]

Grade IIIA (classic HG) (Figure 5A,B) was characterized by marked architectural disorganization with loss of polarity, fused papillae and/or solid areas, and complete absence of umbrella cells. Cytological atypia was severe, including large pleomorphic nuclei (>5× lymphocyte size), diffuse hyperchromasia, prominent nucleoli and loss of nuclear grooves. Mitotic figures were frequent, including atypical mitotic figures, with frequent apoptosis. These features represented fully developed conventional HG urothelial carcinoma morphology.

**Figure 5:**
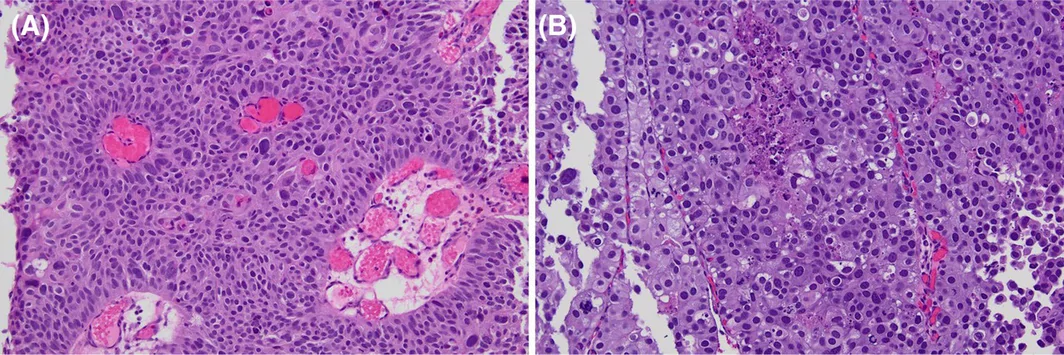
Grade IIIA (Classic High‐Grade) papillary urothelial carcinoma. **(A)** Marked architectural disorganisation with loss of cellular polarity and marked pleomorphism. **(B)** High‐power view demonstrating severe cytological atypia with large pleomorphic nuclei (>5× lymphocyte size), diffuse hyperchromasia, frequent mitotic figures and abundant apoptosis and necrosis, representing fully developed conventional high‐grade urothelial carcinoma morphology. [Colour figure can be viewed at wileyonlinelibrary.com]

Grade IIIB (HG with nuclear anaplasia) (Figure 6A,B) exhibited the most severe degree of cytological atypia, including solid growth, complete loss of polarity and cohesion, and marked nuclear anaplasia defined by nuclei greater than four times the size of adjacent tumour nuclei. Tumours demonstrated profound pleomorphism, frequent atypical mitotic figures, extensive apoptosis and commonly associated necrosis. This category represented the extreme end of HG cytological atypia.

**Figure 6:**
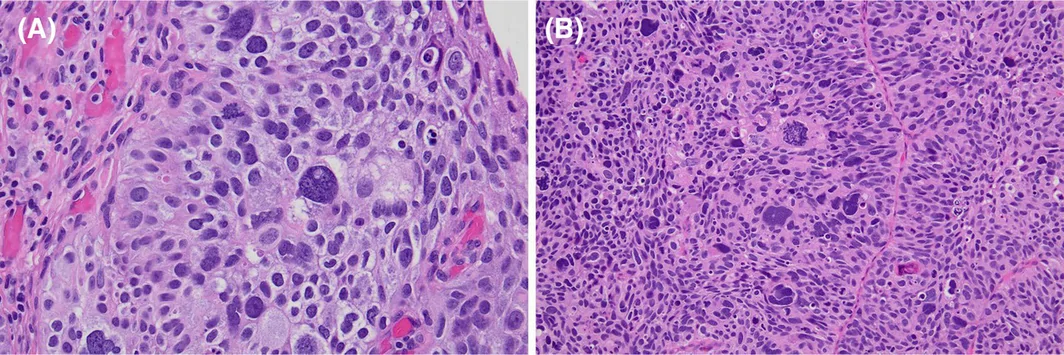
Grade IIIB (High‐Grade with Nuclear Anaplasia) papillary urothelial carcinoma. **(A, B)** High‐power view demonstrating the most severe degree of cytological atypia, with marked nuclear anaplasia characterized by nuclei greater than four times the size of adjacent tumour nuclei, profound pleomorphism, complete loss of polarity and cohesion and frequent atypical mitotic figures, representing the extreme end of high‐grade cytological atypia. [Colour figure can be viewed at wileyonlinelibrary.com]

The five‐tier framework was intended to assess and cross‐examine morphological transitions along the biological spectrum of non‐invasive papillary urothelial carcinoma rather than imply the existence of five clinically distinct management categories. For comparison with established grading systems, Grade I corresponded to WHO 1973 G1, while Grade IIIA corresponded to WHO 1973 G3. Intermediate categories (IIA and IIB) represented transitional morphological spectra between low‐ and high‐grade diseases supported by previously described hybrid grading models. For comparative analyses, Grades I and IIA were grouped as LG within the WHO 2004/2016 framework, Grade IIB corresponded to HG‐G2 in the hybrid three‐tier system and Grades IIIA/IIIB corresponded to HG‐G3.

All slides were reviewed jointly by two urological pathologists (J.K.N. and R.A.) without knowledge of original pathology diagnosis/grade or clinical outcomes. Interobserver agreement analysis was not the primary aim of this study. Grading was assigned in real time by mutual agreement using the predefined study criteria developed for the continuous morphological grading framework. Final classification was based on the highest grade component (irrespective of extent) identified while preserving the conceptual framework of a continuous morphological spectrum.

### Study Outcomes

The primary endpoints of the study were recurrence, grade progression and stage progression. Recurrence was classified as any disease reappearance, post 90 days initial resection. Grade progression is defined as any increase in grade to HG, stage progression was defined as any increase in T stage from Ta to T1 or higher (histologically confirmed) including lymph node metastasis or distant metastasis, excluding events on re‐resection within 90 days.

Time to progression was measured from the date of TURBT, with patients who did not experience progression censored at their last follow‐up. Patients were followed until their last clinical visit, progression or death.

### Statistical Analysis

Patient demographics and baseline clinicopathological characteristics were summarized using descriptive statistics. Continuous variables were compared using the Wilcoxon rank‐sum test or Kruskal–Wallis test, as appropriate, and categorical variables were compared using the chi‐squared test or Fisher exact test when expected cell counts were small. Data were reported as mean ± standard deviation (SD) or counts with percentages, as appropriate.

The prognostic performance of the evaluated grading systems for recurrence and T1+ stage progression was assessed using receiver operating characteristic (ROC) curve analysis and time‐to‐event methods. Discriminative ability was quantified using the area under the ROC curve (AUC) with 95% confidence intervals (CIs). Pairwise comparisons of AUCs were performed using bootstrap resampling with 10,000 iterations based on the common complete‐case dataset used for all grading systems.

Time‐to‐event analyses were performed using Cox proportional hazards regression models for recurrence and T1+ stage progression. Univariable models were constructed for each grading system using the lowest grade category as the reference group. Hazard ratios (HRs) with 95% CIs were reported. The proportional hazards assumption was assessed using Schoenfeld residuals.

To compare overall model‐performance, likelihood‐ratio (LR) chi‐square statistics, Akaike information criterion (AIC), Bayesian information criterion (BIC) and Harrell's concordance index (C‐index) with 95% CIs were calculated for each Cox model. Lower AIC and BIC values were interpreted as indicating a better balance between model fit and complexity, whereas higher C‐index values indicated greater discriminative ability.

All statistical analyses were performed using R software (version 4.6.0). All tests were two‐sided and a *P*‐value <0.05 was considered statistically significant.

## Results

### Search Strategy and Histomorphological Reclassification

The PIRO search strategy initially identified 1,733 patients diagnosed with non‐invasive papillary urothelial carcinoma, including 702 LG, 173 predominantly LG with focal HG and 858 HG tumours according to original pathology reports (Figure 7). After application of study inclusion criteria, 494 LG, 49 predominantly LG with focal HG and 399 HG cases were excluded. Histological material was unavailable for pathology re‐review in an additional 106 LG and 213 HG cases. The final study cohort consisted of 472 patients with available tissue for centralized pathology review who met all study inclusion criteria, including 102 LG, 124 predominantly LG with focal HG and 246 HG tumours (Figure 7).

**Figure 7:**
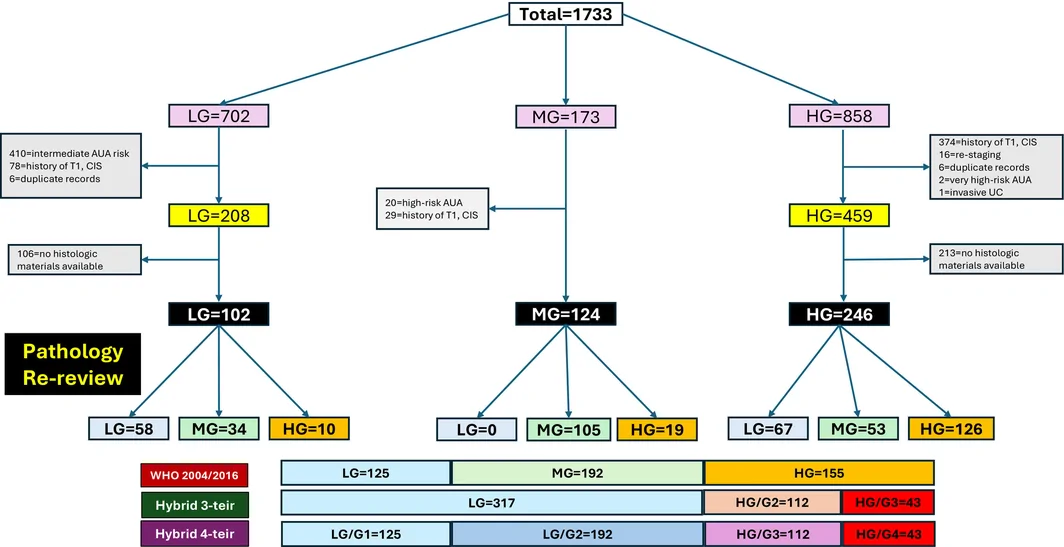
Flow diagram of cohort selection and histopathological reclassification and grading of non‐invasive papillary urothelial carcinoma cases (*n* = 1733). MG, mixed grade (non‐invasive low‐grade papillary urothelial carcinoma with a focal high‐grade component). [Colour figure can be viewed at wileyonlinelibrary.com]

Following joint histopathological review using the predefined investigational framework, all tumours were reassigned into one of five morphological categories representing a continuous spectrum of architectural, cytological and proliferative atypia (Figure 7). Reclassification demonstrated progressive transitions between conventional LG and HG morphology rather than discrete binary separation, with intermediate categories capturing tumours showing incremental deviations in polarity, cohesion, nuclear atypia, mitotic activity and apoptotic burden.

### Clinicopathological Characteristics across the Morphological Spectrum

Table 2 summarizes demographic, clinicopathological and oncological outcome characteristics stratified according to the investigational five‐tier framework. The overall mean patient age was 71.9 years, and 75.8% (358/472) of patients were male. Mean tumour size was 2.8 cm, and 39.4% (186/472) of tumours were multifocal.

**Table 2:** Clinicopathological characteristics and oncological outcomes across the investigational five‐tier morphological spectrum of non‐invasive papillary urothelial carcinoma (*n* = 472)

	Total (*n* = 472)	I (*n* = 125)	IIA (*n* = 192)	IIB (*n* = 112)	IIIA (*n* = 36)	IIIB (*n* = 7)	*P*‐value
Age, year (mean ± SD)	71.9 ± 11.7	70.8 ± 11.8	71.2 ± 11.5	73.9 ± 12.2	73.0 ± 10.8	72.3 ± 11.9	0.152
Sex (male %)	358 (75.8%)	95 (76.0%)	144 (75.0%)	88 (78.6%)	26 (72.2%)	5 (71.4%)	0.90
Tumour size, cm (mean ± SD)	2.8 ± 1.7	2.3 ± 1.2	3.0 ± 1.9	2.9 ± 1.8	3.3 ± 1.7	4.3 ± 2.5	0.006
Focality (multifocal %)	186 (39.4%)	27 (21.6%)	83 (43.2%)	54 (48.2%)	20 (55.6%)	2 (28.6%)	<0.001
Follow‐up time (months, mean ± SD)	41.8 ± 28.3	40.5 ± 28.4	46.0 ± 31.0	36.7 ± 24.8	38.4 ± 21.6	44.5 ± 19.6	0.155
Recurrence, *n* (%)	168 (35.6%)	35 (28.0%)	66 (34.4%)	42 (37.5%)	21 (58.3%)	4 (57.1%)	0.011
Recurrence time (months, mean ± SD)	16.3 ± 17.2	18.3 ± 20.5	15.8 ± 16.8	15.2 ± 14.8	13.2 ± 12.3	34.9 ± 32.1	0.371
Progression (any), *n* (%)	51 (10.8%)	3 (2.4%)	25 (13.0%)	6 (5.4%)	13 (36.1%)	4 (57.1%)	<0.0001
Grade progression (%)	23 (4.9%)	2 (1.6%)	21 (10.9%)	N/A	N/A	N/A	
T1 stage progression (%)	17 (3.6%)	1 (0.8%)	3 (1.6%)	4 (3.6%)	7 (19.4%)	2 (28.6%)	
T2 stage progression (%)	5 (1.1%)	0 (0%)	0 (0%)	2 (1.8%)	3 (8.3%)	0 (0%)	
Metastatic progression (%)	4 (0.8%)	0 (0%)	1 (0.5%)	0 (0%)	3 (8.3%)	0 (0%)	
Progression time (months, mean ± SD)	21.5 ± 21.9	28.4 ± 20.2	19.8 ± 22.5	26.8 ± 26.2	15.4 ± 13.1	56.8 ± 33.9	0.423

NA, not applicable.

Overall recurrence occurred in 35.6% (168/472) of patients, whereas grade progression was observed in 7.2% (23/317 excluding HG tumours) and stage progression occurred in 5.5% (26/472). Specifically, progression to T1 disease occurred in 3.6% (17/472), progression to T2 disease in 1.1% (5/472) and metastatic progression in 0.8% (4/472). Mean time to recurrence and progression was 16.3 and 21.5 months, respectively.

No statistically significant differences in age (range 70.8–73.9 years; *P* = 0.152) or sex distribution (male proportion 71.4%–78.6%; *P* = 0.90) were observed across the morphological categories. In contrast, tumour size increased progressively across the grading continuum, ranging from 2.3 cm in Grade I to 4.3 cm in Grade IIIB (*P* = 0.006). Multifocality similarly demonstrated a strong association with increasing morphological atypia, increasing from 21.6% in Grade I tumours to 55.6% in Grade IIIA tumours (*P* < 0.001).

Oncological outcomes also demonstrated a progressive biological gradient across the morphological spectrum. Recurrence rates increased incrementally from 28.0% in Grade I tumours to 58.3% in Grade IIIA and 57.1% in Grade IIIB tumours (*P* = 0.011). Similarly, progression risk demonstrated a marked grade‐dependent increase, particularly within Grades IIIA and IIIB (*P* < 0.0001). Importantly, all forms of progression, including grade progression, T1 progression, T2 progression and metastatic progression, were disproportionately concentrated within the upper morphological spectrum, particularly Grades IIIA and IIIB (Table 2). Although progression time did not differ significantly between groups (*P* = 0.423), numerically shorter progression intervals were observed in Grade IIIA tumours.

### Prognostic Performance of Conventional and Hybrid Grading Systems for Tumour Recurrence

Tumour recurrence occurred in 168 of 472 patients (35.6%). Univariable Cox regression demonstrated increasing recurrence risk across progressively advanced morphological categories within each grading system (Table 3). Within the WHO 2004/2016 classification, HG tumours were associated with increased recurrence risk compared with LG tumours (HR 1.56, 95% CI 1.15–2.13; *P* = 0.005).

**Table 3:** Univariable cox regression analysis of conventional and hybrid grading systems for recurrence and T1+ progression

Variable/level	Recurrence		T1+ progression	
	HR (95% CI)	*P*‐value	HR (95% CI)	*P*‐value
*WHO 2004/2016*				
LG	—	—		
HG	1.56 (1.15, 2.13)	0.005	9.97 (3.74, 26.58)	<0.001
*Hybrid Three‐Tier*				
LG	—	—		
HG‐G2	1.36 (0.95, 1.96)	0.092	3.94 (1.20, 12.96)	0.024
HG‐G3	2.07 (1.34, 3.22)	0.001	26.09 (9.41, 72.31)	<0.001
*Hybrid Four‐Tier*				
LG‐G1	—	—		
LG‐G2	1.22 (0.81, 1.83)	0.349	2.43 (0.27, 21.78)	0.427
HG‐G3	1.54 (0.98, 2.42)	0.058	7.44 (0.89, 61.93)	0.063
HG‐G4	2.35 (1.40, 3.92)	0.001	49.28 (6.49, 373.98)	<0.001
*Investigational Five‐Tier*				
I	—	—		
IIA	1.22 (0.81, 1.83)	0.349	2.43 (0.27, 21.76)	0.427
IIB	1.54 (0.98, 2.42)	0.058	7.46 (0.90, 62.11)	0.063
IIIA	2.53 (1.47, 4.35)	<0.001	54.07 (7.04, 415.00)	<0.001
IIIB	1.70 (0.61, 4.80)	0.313	31.78 (2.88, 350.78)	0.005

Univariable Cox proportional hazards models were fit separately for recurrence (*N* = 472; 168 events) and T1+ progression (*N* = 472; 26 events). The lowest grade category served as the reference level for each grading system.

CI, confidence interval; HR, hazard ratio.

Within the Hybrid 3‐Tier system, HG‐G3 tumours demonstrated significantly increased recurrence risk (HR 2.07, 95% CI 1.34–3.22; *P* = 0.001), whereas HG‐G2 tumours showed a more modest association (HR 1.36, 95% CI 0.95–1.96; *P* = 0.092). Similar trends were observed in the Hybrid 4‐Tier and Investigational 5‐Tier systems, with progressively higher grades generally associated with increasing recurrence risk.

Despite these associations, overall discriminative performance for recurrence was modest across all grading systems (Table 4A). C‐indices ranged from 0.544 for WHO 2004/2016 to 0.563 for the Hybrid 4‐Tier and Investigational 5‐Tier systems. Likelihood‐ratio statistics increased modestly with greater morphological stratification, but differences in AIC and BIC were small. The Hybrid 3‐Tier model demonstrated the lowest AIC (1894.5), suggesting the most favourable balance between model fit and complexity for predicting recurrence.

**Table 4:** Model‐performance of conventional and hybrid grading systems for prediction of recurrence and T1+ progression

A. Recurrence (*N* = 472; events = 168)						
Model	LR χ^2^	Df	LR *P*‐value	AIC	BIC	C‐index (95% CI)
WHO 2004/2016	7.67	1	0.006	1895.1	1898.2	0.544 (0.506, 0.582)
Hybrid 3‐Tier	10.31	2	0.006	1894.5	1900.7	0.549 (0.510, 0.589)
Hybrid 4‐Tier	11.21	3	0.011	1895.6	1904.9	0.563 (0.520, 0.605)
Investigational 5‐Tier	11.78	4	0.019	1897.0	1909.5	0.563 (0.521, 0.606)

B. Progression T1+ (*N* = 472; events = 26)						
Model	LR χ^2^	Df	LR *P*‐value	AIC	BIC	C‐index (95% CI)
WHO 2004/2016	28.73	1	<0.001	266.1	267.3	0.745 (0.660, 0.829)
Hybrid 3‐Tier	46.24	2	<0.001	250.6	253.1	0.806 (0.707, 0.904)
Hybrid 4‐Tier	46.98	3	<0.001	251.8	255.6	0.814 (0.717, 0.912)
Investigational 5‐Tier	47.53	4	<0.001	253.3	258.3	0.818 (0.719, 0.917)

Lower AIC/BIC values indicate better model fit after accounting for model complexity; higher C‐index values indicate greater discriminative ability.

AIC, Akaike information criterion; BIC, Bayesian information criterion; C‐index, Harrell's concordance index; LR χ^2^, likelihood‐ratio chi‐square statistic.

### Prediction of T1+ Stage Progression

T1+ progression occurred in 26 of 472 patients (5.5%). ROC analysis demonstrated improved discrimination with increasing morphological stratification (Figure 8). The WHO 2004/2016 system achieved an AUC of 0.754, whereas the Hybrid 3‐Tier, Hybrid 4‐Tier and Investigational 5‐Tier systems achieved AUCs of 0.815, 0.828 and 0.827, respectively. Pairwise comparisons demonstrated significantly improved discrimination for all tiered systems compared with WHO 2004/2016 (all *P* < 0.001), whereas differences among the three tiered systems were not statistically significant.

**Figure 8:**
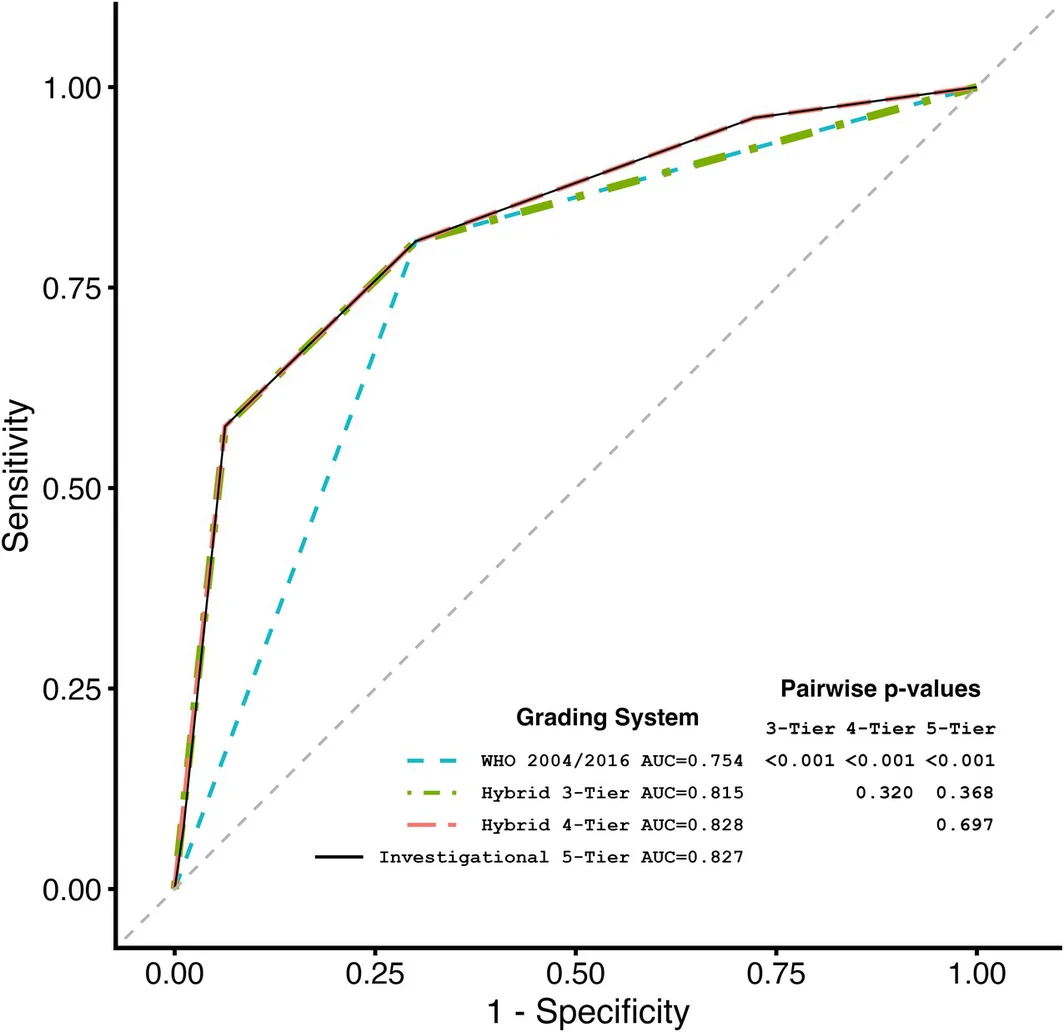
Receiver operating characteristic analysis of conventional and hybrid grading systems for prediction of T1+ stage progression. [Colour figure can be viewed at wileyonlinelibrary.com]

Univariable Cox regression analysis similarly demonstrated progressively increasing risk of progression across increasingly advanced morphological categories (Table 3). Within the WHO 2004/2016 system, HG tumours were associated with a markedly increased risk of progression compared with LG tumours (HR 9.97, 95% CI 3.74–26.58; *P* < 0.001).

Within the Hybrid 3‐Tier system, HG‐G2 tumours demonstrated increased progression risk (HR 3.94, 95% CI 1.20–12.96; *P* = 0.024), whereas HG‐G3 tumours demonstrated substantially higher risk (HR 26.09, 95% CI 9.41–72.31; *P* < 0.001). Similar stepwise increases were observed within the Hybrid 4‐Tier and Investigational 5‐Tier systems. The highest risk was observed for Grade IIIA within the Investigational 5‐Tier system (HR 54.07, 95% CI 7.04–415.00; *P* < 0.001).

Model‐performance analyses corroborated the ROC findings (Table 4B). Likelihood ratio chi‐square statistics increased from 28.73 for WHO 2004/2016 to 46.24, 46.98 and 47.53 for the Hybrid 3‐Tier, Hybrid 4‐Tier and Investigational 5‐Tier systems, respectively. Concordance indices similarly improved from 0.745 to 0.806, 0.814 and 0.818. Although the Investigational 5‐Tier system demonstrated the highest concordance, the Hybrid 3‐Tier model achieved the lowest AIC (250.6) and BIC (253.1), indicating the most favourable balance between prognostic performance and model complexity.

## Discussion

Bladder cancer grading systems have undergone multiple refinements over the past century, reflecting continuous efforts to improve diagnostic consistency and prognostic accuracy (Figure 9).^4^, ^6^, ^7^, ^8^, ^9^, ^10^, ^11^, ^12^, ^13^ Early systems, such as Broders^12^ (1922) and Franksson^11^ (1950), relied on subjective assessments of cellular anaplasia and atypia, while subsequent classifications introduced more structured criteria‐based on cellular differentiation and architectural disarray (e.g. Bergkvist *et al*.^10^ 1965; WHO 1973^14^). A major conceptual shift occurred with the WHO/ISUP 1999^13^ and WHO 2004^15^ classifications, which incorporated the category of PUNLMP and ultimately established the widely used system of PUNLMP, LG and HG tumours, further refined in later WHO updates (2016, 2022).^16^, ^20^ These systems aimed to standardize terminology while linking histological grade more closely to clinical outcomes. Both WHO 1973 and WHO 2004/2016 grading systems demonstrate important but distinct limitations in the prognostic stratification of non‐invasive papillary urothelial carcinoma. While WHO 1973 provides broader capture of progression risk, it is limited by substantial intra‐category heterogeneity, particularly within the G2 group. In contrast, WHO 2004 offers improved histological reproducibility but shows reduced prognostic resolution, especially within the HG category, where biologically diverse tumours are grouped together.^5^, ^34^, ^35^, ^36^

**Figure 9:**
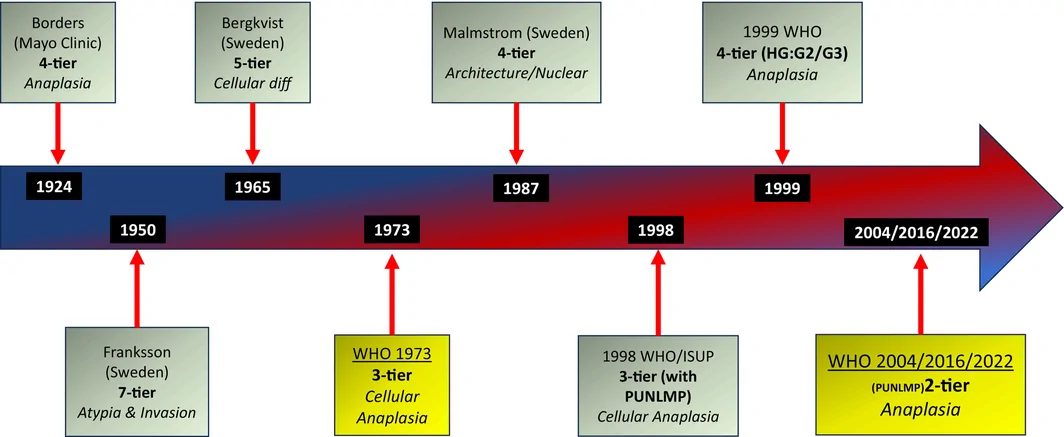
Historical evolution of bladder cancer grading systems. [Colour figure can be viewed at wileyonlinelibrary.com]

More recently, efforts have focused on optimizing prognostic stratification while maintaining reproducibility. A four‐tier system was proposed by Cheng *et al*.^7^ (2012), while alternative three‐tier approaches were suggested by van der Kwast *et al*.^4^ (2021) and reinforced by the International Society of Urological Pathology (ISUP) in 2022,^6^ all emphasizing clinically relevant subclassification of HG disease. The hybrid three‐tier grading system combines features of the WHO 1973 and WHO 2004 classifications by retaining WHO 2004 LG tumours (including PUNLMP) as a single category and subdividing HG tumours into G2 and G3.^4^ Although PUNLMP was not included in the present cohort because the study focused exclusively on non‐invasive papillary urothelial carcinoma, PUNLMP would be expected to occupy the lowest end of the proposed morphological continuum, corresponding most closely to Grade I.^21^, ^22^ We do not propose replacing the WHO diagnostic category of PUNLMP but rather view it as representing the least biologically aggressive end of the continuum. This interpretation is also consistent with current clinical guidelines, in which PUNLMP and low‐grade papillary urothelial carcinoma are managed similarly.^18^, ^37^

The present study was designed not only to compare contemporary grading systems, but also to further investigate the histomorphological continuum underlying non‐invasive papillary urothelial carcinoma. Rather than viewing tumour grade as a series of discrete categorical states, our findings support the concept that non‐invasive papillary urothelial carcinoma exists along a progressive biological spectrum characterized by cumulative architectural disorganization, increasing cytological atypia, loss of polarity, nuclear pleomorphism, chromatin abnormalities, mitotic activity and apoptotic change. This interpretation aligns closely with recent contemporary reviews and hybrid grading proposals emphasizing the continuous nature of bladder cancer biology rather than rigid binary separation.^3^, ^26^, ^31^, ^38^

Several important observations emerged from our analyses. First, recurrence and progression demonstrated fundamentally different relationships with histological grade. Across all evaluated grading systems, recurrence prediction remained poor, with uniformly limited discriminative performance and ROC curves approximating chance levels. These findings are highly consistent with prior literature demonstrating that existing grading systems and established NMIBC risk models show substantially weaker performance for recurrence prediction than for progression prediction.^1^, ^17^, ^39^ Soukup *et al*. in the EAU systematic review of WHO grading systems, concluded that currently available grading classifications remain suboptimal for recurrence prediction and failed to demonstrate clear superiority of the WHO 2004/2016 system over the WHO 1973 system.^2^ Similarly, Ferro *et al*. demonstrated markedly lower c‐index values for recurrence prediction (0.56–0.61) compared with progression prediction (0.74–0.81) across conventional and hybrid grading systems, highlighting the biological divergence between recurrence and progression risk assessment.^40^ Jahnson *et al*. further showed that even combined four‐tier grading systems provide only modest improvements in recurrence discrimination.^41^

Importantly, these observations likely reflect the fundamentally multifactorial biology underlying NMIBC recurrence. Unlike stage progression, which is more directly linked to intrinsic tumour aggressiveness and biological potential, recurrence is strongly influenced by urothelial field instability, multifocal tumourigenesis, incomplete transurethral resection, tumour implantation and re‐seeding, intravesical treatment response and host microenvironmental factors.^17^, ^37^ Teoh *et al*. summarized several mechanisms contributing to NMIBC recurrence, including incomplete tumour resection, occult lesions, intraluminal tumour seeding and field cancerization.^42^ Molecular and genomic studies further support this concept. Hafner *et al*.^43^ demonstrated evidence for both intraluminal tumour seeding and oligoclonal tumour development arising from field effects. More recently, Strandgaard *et al*.^44^ identified driver mutations and immune exhaustion signatures within morphologically normal urothelium associated with subsequent tumour development, while Marzouka *et al*.^45^ showed that recurrent tumours often harbour genomic rearrangements inconsistent with direct tumour‐to‐tumour derivation, supporting a persistent evolving field effect model rather than simple local recurrence.

Accordingly, histological grade appears to contribute only modestly to recurrence risk when assessed in isolation. Contemporary clinical guidelines similarly emphasize that recurrence risk in NMIBC depends on multiple interacting clinicopathological variables rather than grade alone.^18^ Importantly, although recurrence remains clinically relevant because of its impact on surveillance burden, procedural morbidity, patient anxiety and healthcare costs, disease stage progression carries substantially greater prognostic and therapeutic significance in routine clinical practice.^5^, ^17^, ^37^, ^39^ Progression to invasive disease remains the principal determinant of cancer‐specific morbidity, mortality, need for radical treatment and long‐term oncological outcome. Consistent with this concept, our analyses demonstrated substantially stronger discrimination and biological stratification for T1+ progression than for recurrence across all evaluated grading systems, supporting the concept that histological grade primarily reflects invasive and biological progression potential rather than recurrence propensity.

In contrast to recurrence, progression to T1+ disease demonstrated a substantially stronger and more biologically coherent relationship with histological grade. Across all evaluated grading systems, discriminatory performance for progression was consistently superior to that observed for recurrence, supporting the concept that histological grading primarily reflects invasive and biological progression potential rather than recurrence propensity alone. These findings are highly concordant with prior studies demonstrating that histological grade is one of the most important determinants of progression risk in NMIBC. Soukup *et al*. showed that grade remains consistently and strongly associated with progression across multiple cohorts.^2^ Similarly, Chen *et al*. demonstrated that the WHO 2004/2016 classification significantly predicted progression on multivariable analysis while failing to predict recurrence, further emphasizing the biological divergence between these outcomes.^46^ Ferro *et al*. likewise showed substantially higher c‐index values for progression prediction (0.74–0.81) than for recurrence prediction (0.56–0.61) across conventional and hybrid grading systems, findings that closely parallel the results of the present study.^40^

Our findings further suggest that increasing morphological refinement improves prognostic stratification for T1+ progression. Compared with the WHO 2004/2016 system, the hybrid grading systems demonstrated superior discriminatory performance, with higher AUCs, likelihood‐ratio statistics and concordance indices. Notably, most of this improvement was achieved with the Hybrid 3‐Tier system, whereas further subdivision into four or five tiers provided only modest incremental gains despite increased model complexity. These observations are consistent with emerging evidence supporting hybrid grading approaches that retain the biological strengths of the WHO 1973 system while preserving the clinical practicality and reproducibility of the WHO 2004/2016 framework.^1^, ^2^, ^22^, ^25^, ^30^, ^47^

In the large EAU individual patient analysis of 5145 NMIBC cases, both WHO 1973 and WHO 2004 independently predicted progression, with WHO 1973 demonstrating stronger prognostic performance.^1^ Importantly, hybrid four‐tier stratification improved Harrell's concordance index beyond either system alone and identified substantially different progression risks within morphologically heterogeneous WHO 2004 HG tumours.^1^ Specifically, 5‐year progression rates increased progressively from LG‐G1 (1.4%) and LG‐G2 (3.8%) to HG‐G2 (7.7%) and HG‐G3 (18.8%), demonstrating that biologically meaningful progression thresholds exist within conventionally grouped high‐grade disease.^1^

These observations were subsequently validated by Downes *et al*., who systematically compared WHO 1973, WHO 2004 and hybrid grading systems in a cohort of 609 dual‐graded NMIBC cases with long‐term follow‐up.^26^ Both hybrid three‐tier and four‐tier systems demonstrated superior prognostic performance for progression‐free and event‐free survival compared with either WHO 1973 or WHO 2004 alone, with higher concordance indices and AUC values.^26^ Marked differences in progression were particularly observed within the conventional WHO 2004 HG category, supporting substantial biological heterogeneity among HG tumours.^26^ In contrast, LG subgroups showed relatively similar behaviour, leading the authors to propose maintaining the WHO 2004 LG category while subdividing HG tumours into biologically distinct prognostic groups.^26^ These findings closely parallel the present study, in which the greatest prognostic separation was likewise observed within the upper morphological spectrum, particularly between Grades IIIA and IIIB.

Further support for this concept was provided by the largest contemporary study by Beijert *et al*. evaluating hybrid grading performance, which included 9712 primary Ta/T1 NMIBC cases across three independent cohorts.^25^ Importantly, subgroup analyses demonstrated that the prognostic value of the hybrid grading system was maintained within Ta tumours alone.^25^ The authors employed a simplified hybrid three‐tier model that retained the WHO 2004 low‐grade category while subdividing HG tumours into HG‐G2 and HG‐G3 categories. Across all cohorts, progression rates at 5 years were consistently higher for HG‐G3 tumours (18%–20%) compared with HG‐G2 tumours (7%–9%), with hybrid grading outperforming WHO 2004 alone in prognostic discrimination.^25^ These findings support the concept that clinically meaningful biological heterogeneity is concentrated within the conventional HG category and that separating HG‐G2 from HG‐G3 may improve risk stratification and therapeutic decision‐making.

In a separate study, Beijert *et al*. specifically examined the prognostic significance of HG‐G3 tumours in a large multicentre cohort of 5170 primary Ta–T1 bladder tumours from 17 institutions.^47^ HG‐G3 tumours represented a relatively uncommon subset, accounting for 7.5% of all Ta–T1 tumours, and 42% were classified as intermediate risk under the updated EAU NMIBC risk stratification system.^47^ While recurrence rates were similar between HG‐G3 and HG‐G2 tumours, progression outcomes differed substantially, with 5‐year progression rates of 3.6% for HG‐G2 and 13% for HG‐G3 tumours.^47^ Importantly, time to progression for HG‐G3 tumours was significantly shorter than for HG‐G2 tumours (*P* < 0.001), supporting the concept that HG‐G3 tumours represent a biologically distinct higher risk subgroup within NMIBC.^47^ These findings support a more refined approach to risk assessment rather than uniformly categorizing all HG‐G3 tumours as high risk, and further reinforce refinement of the conventional WHO 2004 high‐grade category into clinically meaningful prognostic subgroups.

The present findings also align with emerging molecular models of urothelial carcinogenesis. Low‐grade papillary tumours are frequently associated with *FGFR3* alterations and relatively stable luminal‐papillary molecular phenotypes, whereas HG tumours more commonly demonstrate *TP53* pathway alterations, chromosomal instability, increased proliferative activity and progression‐associated basal or genomically unstable features.^48^, ^49^ These observations further support the concept that morphological progression in NMIBC reflects an underlying biological continuum and suggest that hybrid grading systems may more closely parallel the molecular evolution of urothelial neoplasia than binary classification alone.

Importantly, the Grade IIA category should not be interpreted as equivalent to the WHO 2022 category of predominantly low‐grade papillary urothelial carcinoma with a focal (<5%) high‐grade component. Rather, these intermediate lesions appear to represent progressive stages of architectural and cytological evolution preceding the emergence of unequivocal high‐grade differentiation. This interpretation is supported by molecular and clinicopathological studies demonstrating that tumours with focal high‐grade components often harbour progression‐associated proliferative and genomic alterations, suggesting that overt high‐grade morphology represents a later stage along a continuous biological spectrum.^31^, ^32^, ^33^ Accordingly, Grade IIA was intended to capture early transitional changes within the low‐grade pathway rather than grade‐heterogeneous tumours containing bona fide high‐grade morphology.

An additional point deserving clarification concerns the distinction between Grade IIIB and giant cell urothelial carcinoma. Although the marked nuclear pleomorphism and anaplasia observed in Grade IIIB tumours may show morphological overlap with giant cell urothelial carcinoma, all Grade IIIB tumours in the present study were conventional non‐invasive papillary urothelial carcinomas. Grade IIIB represents the extreme end of the morphological continuum of high‐grade non‐invasive papillary urothelial carcinoma rather than a distinct diagnostic entity. In contrast, giant cell urothelial carcinoma is, by definition, an invasive subtype of urothelial carcinoma.^16^ The present study further extends these observations by demonstrating that progressive architectural disorganization, increasing cytological atypia, loss of polarity and proliferative activity are associated with progressively increasing risk of invasive progression. The hybrid grading systems identified biologically distinct subsets within conventionally defined high‐grade tumours and provided improved prognostic discrimination compared with the binary WHO 2004/2016 classification. Although additional subdivision beyond the Hybrid 3‐Tier system yielded only modest improvements in model‐performance, the four‐tier and investigational five‐tier frameworks further illustrated the morphological continuum underlying NMIBC progression. Rather than defining multiple clinically actionable grade categories, these findings suggest that progression risk becomes progressively enriched along a spectrum of adverse morphological features. Overall, the results support the broader concept that bladder cancer grading may be more accurately viewed as a continuum of biological progression than as a strictly dichotomous process.

A major strength of the present study is the centralized slide‐level review of all cases by dedicated urological pathologists using predefined morphological criteria and blinded outcome assessment. This approach minimized variability related to original diagnostic reporting, ensured consistent application of grading parameters across all evaluated systems, and enabled detailed characterization of morphological variation across the disease spectrum.

Beyond improving prognostic stratification, the proposed hybrid grading system may also have important clinical implications. Current management of NMIBC relies heavily on histological grade to guide intravesical therapy, surveillance intensity and consideration of early radical cystectomy.^18^, ^37^ Our findings suggest that progression risk is not uniformly distributed across conventional high‐grade tumours but is concentrated within the upper end of the morphological spectrum, particularly Grades IIIA and IIIB. If validated prospectively, this refined stratification could facilitate more individualized management. Patients with lower risk morphology may be suitable for treatment de‐escalation and less intensive surveillance, whereas those at highest risk for progression may be candidates for treatment intensification. In this context, the highest risk morphological groups may represent a population of particular interest for emerging first‐line BCG‐based combination strategies, such as durvalumab plus BCG, which improved disease‐free survival in BCG‐naïve high‐risk disease in the phase 3 POTOMAC trial,^50^ or other intensified immunotherapy approaches currently under prospective evaluation (e.g. nogapendekin alfa inbakicept plus BCG in QUILT‐2.005), as well as for earlier consideration of radical cystectomy or enrollment in clinical trials of novel bladder‐preserving strategies. Importantly, because treatment outcomes were not evaluated in the present study, these implications should be considered hypothesis‐generating and require prospective validation before influencing clinical practice.

Several limitations should be acknowledged. The study was retrospective and derived from a single tertiary referral centre, introducing the potential for selection bias. Formal interobserver reproducibility analysis was not performed as a primary aim of the current investigation; all cases instead underwent centralized, blinded consensus review by two urological pathologists using predefined grading criteria, which minimizes but does not eliminate potential systematic grading bias. The absence of quantitative reproducibility data are a recognized limitation, particularly for the investigational five‐tier framework. It should be noted that this challenge is not unique to the present framework as published literature demonstrates only moderate interobserver agreement for existing WHO 2004/2016 and WHO 1973 systems (κ approximately 0.41–0.60), whereas hybrid three‐tier grading has achieved substantially higher agreement among international uropathologists (κ 0.62–0.68) in dedicated reproducibility studies.^3^, ^30^ Whether the more refined distinctions of the five‐tier framework are similarly reproducible requires formal assessment and represents a priority for future investigation. In addition, the investigational grading framework requires external validation and assessment in independent cohorts before broader clinical application. Finally, the number of progression events was relatively limited, resulting in wide confidence intervals for some higher grade categories and limiting the precision of risk estimates.

Despite these limitations, the present study demonstrates that hybrid grading systems improve progression‐risk stratification compared with the binary WHO 2004/2016 classification while better capturing the biological heterogeneity within conventionally defined high‐grade NMIBC. The findings further suggest that much of the prognostic benefit can be achieved through selective refinement of the high‐grade spectrum, as reflected by the strong performance of the Hybrid 3‐Tier system. These results support continued refinement of grading paradigms towards biologically informed hybrid approaches that more closely align histomorphology with tumour behaviour and clinically meaningful progression risk.

## Conclusions

Histological grading in non‐invasive papillary urothelial carcinoma is better conceptualized as a biological and morphological continuum than as a strictly binary process. The WHO 2004/2016 classification, while improving reproducibility and clinical practicality, incompletely captures the heterogeneity within conventionally grouped high‐grade tumours and provides limited prognostic resolution for progression risk.

In this cohort, hybrid grading systems demonstrated superior discrimination for T1+ stage progression compared with WHO 2004/2016, with the Hybrid 3‐Tier model achieving the most favourable balance between prognostic performance and model complexity. Further subdivision into four or five tiers provided only modest incremental gains. In contrast, all grading systems showed limited discriminatory ability for recurrence, consistent with the multifactorial biology underlying NMIBC recurrence that extends beyond tumour morphology alone.

These findings support selective refinement of the high‐grade spectrum as a practical and biologically informed approach to improving progression‐risk stratification in NMIBC. Future multicentre studies incorporating formal reproducibility assessment, external validation and molecular correlates are warranted to establish optimal grading thresholds and advance the integration of morphological and biological risk stratification in clinical practice.

## Author contributions

Reza Alaghehbandan conceived and designed the study. R. Alaghehbandan and Jane K. Nguyen performed centralized histopathological review and case reclassification. Jianbo Li performed the statistical analysis. Reza Alaghehbandan drafted the manuscript. All authors contributed to data interpretation, critically revised the manuscript for important intellectual content and approved the final version for submission.

## Conflicts of interest

The authors declare no conflict of interest and no funding was obtained for this study.

## Consent

Not applicable. This study used de‐identified, retrospectively collected pathology and clinical data and individual patient consent for publication was not required in accordance with the approving Institutional Review Board's determination. No identifiable patient information is included in this manuscript.

## Data Availability

The data that support the findings of this study are available on request from the corresponding author. The data are not publicly available due to privacy or ethical restrictions.
